# Does predictive accuracy reflect anatomical attention in CVM staging? evidence from explainable held-out vendor evaluation

**DOI:** 10.3389/fmed.2026.1971090

**Published:** 2026-10-14

**Authors:** Yanjun Jiang, Yang Lyu, Fengcong Wang, Ping Liu, Yilan Yang

**Affiliations:** 1 Department of Orthodontics, Ningbo Stomatology Hospital, Ningbo, Zhejiang, China; 2 Engineering Laboratory for Biomaterials and Tissue Regeneration, Ningbo Stomatology Hospital, Ningbo, Zhejiang, China; 3 GreenOne Technology Consulting Co., Ltd, Jinan, Shandong, China; 4 Department of Orthodontics, Jinan Stomatological Hospital, Jinan, Shandong Province, China; 5 Shandong Provincial Health Commission Key Laboratory of Oral Diseases and Tissue Regeneration, Jinan Stomatological Hospital, Jinan, Shandong, China; 6 Shandong Medical and Pharmaceutical University, Jinan, Shandong, China; 7 Department of Orthodontics, HuiZhou Stomatological Hospital, Huizhou, Guangdong, China

**Keywords:** cervical vertebral maturation, clinical reliability, deep learning, explainable artificial intelligence, held-out vendor validation, lateral cephalometric radiographs

## Abstract

**Background:**

Deep learning has shown promising performance for cervical vertebral maturation (CVM) staging, but reported accuracies vary widely across studies. Differences in image preprocessing and imaging systems may affect model performance, while the black-box nature of deep learning makes it unclear whether predictions are based on clinically relevant cervical vertebral morphology.

**Methods:**

A total of 908 lateral cephalometric radiographs from four imaging vendors were retrospectively collected. Of these, 708 images from three vendors were used for model development and internal validation, while 200 images from a held-out vendor were reserved for external testing. ResNet-50 and Swin Transformer were evaluated across six preprocessing pipelines. Classification accuracy, Macro-F1, adjacent-stage accuracy, and severe misclassification were assessed. Grad-CAM and attention rollout were used for qualitative interpretability analysis.

**Results:**

External exact-stage accuracy was consistently lower than internal performance. The highest external accuracies were 0.47 for ResNet-50 with min–max plus *Z*-score normalization and 0.42 for Swin Transformer with min–max plus CLAHE. Adjacent-stage accuracy was substantially higher, but severe misclassifications persisted. Preprocessing effects were architecture-dependent, with no single strategy consistently improving both models. Explainability maps showed variable attention alignment with C2–C4 and occasional emphasis on non-classical anatomical or imaging-dependent regions.

**Conclusions:**

Deep learning performance for CVM staging did not generalize consistently across imaging conditions, and preprocessing influenced different architectures differently. These findings suggest that predictions from machine learning–based CVM models should be interpreted with caution, particularly when applied to data acquired under different imaging conditions.

## 1 Introduction

Accurate identification of the pubertal growth peak is fundamental to orthodontic diagnosis and treatment planning (1). Among the biological indicators used to assess skeletal maturation, cervical vertebral maturation (CVM) has been widely adopted due to its practicality and the routine availability of lateral cephalometric radiographs (2). While the concept was initially introduced to analyze morphological changes of the cervical vertebrae for growth estimation (3, 4), McNamara and Baccetti subsequently refined the method to focus on the second through fourth cervical vertebrae (C2–C4), proposing a six-stage classification system (CVM1–CVM6) (5). This standardized assessment of cervical vertebral morphology remains of substantial clinical importance for optimizing orthodontic intervention (2, 6).

Cervical vertebral maturation reflects the biological progression of skeletal growth during adolescence, particularly the acceleration and deceleration phases of the pubertal growth spurt (2, 5). Changes in vertebral morphology are closely associated with mandibular growth velocity and overall somatic development (1, 7). Clinically, accurate identification of CVM stage enables orthodontists to determine the optimal timing for growth modification therapies, such as functional appliance treatment or orthopedic intervention (8). Initiating treatment too early may result in suboptimal skeletal response (9, 10), whereas delayed intervention may miss the peak growth window, limiting treatment effectiveness (11, 12). Therefore, reliable CVM staging is not merely a radiographic classification task but a critical component of individualized treatment planning.

Recent advances in artificial intelligence (AI), particularly in computer vision, have accelerated the development of automated medical image analysis, including applications in orthodontics (13). Automated CVM staging has attracted increasing attention, as the C2–C4 vertebrae undergo characteristic morphological changes—such as the development of inferior border concavities and shape alterations—that are suitable for data-driven analysis (2, 5). Convolutional neural network (CNN)–based models are well-suited to learn hierarchical representations of local image features (14), while transformer-based architectures can capture long-range contextual dependencies through attention mechanisms. Both architectures offer theoretical advantages for supporting objective, reproducible staging based on expert-annotated reference standards (15). However, published studies applying vision-based neural networks for CVM staging have reported highly variable prediction accuracies, ranging from approximately 57% to 95% (16). This wide dispersion introduces uncertainty regarding clinical reliability. Several factors contribute to these discrepancies, including inadequately reported dataset characteristics (17) and extensive hyperparameter optimization that may reduce robustness on independent clinical data. Furthermore, radiographic images from different vendors may respond inconsistently to preprocessing, leading to heterogeneous effects across network architectures (18). Moreover, most existing approaches function as “black-box” models with limited interpretability (19), restricting clinical trust as it remains unclear whether predictions are driven by meaningful anatomical features or incidental artifacts.

Rather than developing a new classification architecture, this study focused on the interaction between image preprocessing, model architecture, and cross-vendor generalization in CVM staging. Under a unified experimental framework, six image-processing pipelines were systematically evaluated using a CNN-based model (ResNet-50) and a transformer-based model (Swin Transformer), followed by external evaluation using data from a held-out imaging vendor. Architecture-specific XAI methods were further applied to examine whether model attention under different preprocessing conditions was consistent with established morphological criteria for CVM assessment. Through this design, we aimed to provide a systematic assessment of how preprocessing and model architecture influence predictive performance and attention patterns under cross-vendor imaging conditions.

Therefore, this study aimed to: (1) systematically evaluate the effects of different image preprocessing pipelines on CVM staging performance; (2) compare CNN- and transformer-based architectures under identical multi-vendor conditions; and (3) investigate, using explainable AI techniques, whether model attention aligns with established clinical CVM assessment criteria.

## 2 Materials and methods

### 2.1 Study design and ethics

This study was conducted in accordance with the ethical standards outlined in the Declaration of Helsinki (2013 revision). Ethical approval was obtained from the Ethics Committee of the institutional review board. The requirement for informed consent for the general dataset was waived by the institutional review board due to the retrospective nature of the study.

All radiographic data were de-identified prior to analysis. To protect patient privacy, a random identification code was assigned to each subject, and no directly patient-identifiable information (e.g., names and birth dates) was accessible to the investigators. This study was reported in accordance with the Checklist for Artificial Intelligence in Medical Imaging (CLAIM) guidelines (20).

### 2.2 Image dataset

A total of 908 lateral cephalometric images were retrospectively collected from three clinical centers: Ningbo Stomatology Hospital (Ningbo, Zhejiang, China), Jinan Stomatological Hospital (Jinan, Shandong, China), and HuiZhou Stomatological Hospital (Huizhou, Guangdong, China). Imaging systems included Sirona (ORTHOPHOS XG 3D/Ceph, 2000W; full-skull: 2,808 × 2,148 pixels; hemi-skull: 1,804 × 2,148 pixels), NewTom (5G CBCT system; CBCT-derived lateral views; resolution range 2,048 × 768 to 2,001 pixels), Meyer (SS-X10010Dplus; 2,548 × 2,036 pixels), and Vatech (PHT-35LHS; 2,964 × 2,592 pixels). CBCT-derived lateral views were reconstructed to simulate conventional lateral cephalograms and were included to reflect real-world clinical imaging diversity.

To simulate routine clinical conditions, radiographic images were excluded only when minimal diagnostic requirements were not met. The exclusion criteria were as follows: (1). Images in which valid diagnostic information could not be reliably obtained due to acquisition-related issues, such as motion artifacts, ghosting, or severe blurring; (2). Images in which the second to fourth cervical vertebrae (C2–C4) could not be clearly and completely visualized because of anatomical or external occlusion.

All remaining images were included consecutively according to acquisition date, without deliberate preselection. Due to differences in imaging equipment, exposure parameters, operator technique, and patient-specific anatomical variation, substantial heterogeneity was observed across the dataset. Cervical vertebrae exhibited wide variability in angulation, field-of-view coverage, and visibility, with some images clearly depicting C2–C7, whereas others were limited to truncated views encompassing only C2–C4. Differences in imaging system design and acquisition mechanisms further contributed to variability in vertebral appearance and anatomical positioning within the radiographs.

To better reflect practical orthodontic scenarios, images from patients with dental implants, endodontic treatments, and full-coverage crowns were retained rather than excluded. All images were confirmed to originate from distinct individuals, and duplicate radiographs were systematically screened and removed.

Among the 708 images used for internal model development, 440 were acquired using Sirona systems (403 full-skull and 37 hemi-skull images), 150 using NewTom systems, and 118 using Meyer systems. Images acquired using Vatech systems were reserved exclusively for external validation and were not included in the internal training or validation sets. The internal dataset was divided into training and validation subsets using an approximately 80:20 stratified split based on vendor distribution.

In addition, an independent external validation dataset consisting of 200 lateral cephalograms acquired using Vatech systems was collected to assess cross-vendor generalization and real-world robustness. Detailed dataset characteristics are summarized in Table 1.

**Table 1:** Sample distribution and vendor information.

Vendor/Source	Device/Model	Image type	Pixel resolution	Skull coverage	No. of images
Sirona	Sirona Orthophos (ORTHOPHOS XG 3D/Ceph, 2,000 W)	2D Lateral cephalogram	Full-skull: 2,808 × 2,148; Hemi-skull: 1,804 × 2,148 px	Full-skull (*n* = 403), Hemi-skull (*n* = 37)	440
NewTom	NewTom CBCT (5G version FP, 220 Vac ± 10%, 50/60 Hz)	CBCT-derived lateral view	2,048 × (768–2,001) px*	Full-skull	150
Meyer	SS-X10010Dplus (AC220 V, 50 Hz)	2D Lateral cephalogram	2,548 × 2,036 px	Full-skull	118
Vatech	PHT-35LHS, 220 V～, 50 Hz, 0.2 kVA	2D Lateral cephalogram	2,964 × 2,592 px	Full-skull	200

* For NewTom CBCT-derived lateral views, the image width was fixed at 2,048 pixels, while the vertical dimension ranged from 768 to 2,001 pixels depending on the reconstructed field of view.

### 2.3 Ground truth and staging

#### 2.3.1 CVM staging

Cervical vertebral maturation (CVM) staging was performed according to the classification method proposed by McNamara and Baccetti (2, 5). This method is based on evaluation of the presence and progression of concavities along the inferior borders of the second to fourth cervical vertebrae (C2–C4), followed by assessment of morphological changes in the shapes of the third and fourth cervical vertebrae (C3–C4). Under this framework, concavities along the inferior borders of C2–C4 appear sequentially before, during, and after the pubertal growth peak. Concurrently, the shapes of C3 and C4 gradually transition from a trapezoidal configuration in CVM stages 1–3 to a horizontally rectangular shape in CVM4, become square in CVM5, and finally assume a vertically rectangular configuration in CVM6. A schematic and radiographic illustration of these morphological characteristics, adapted from the work of McNamara (2), is shown in Figure 1.

**Figure 1:**
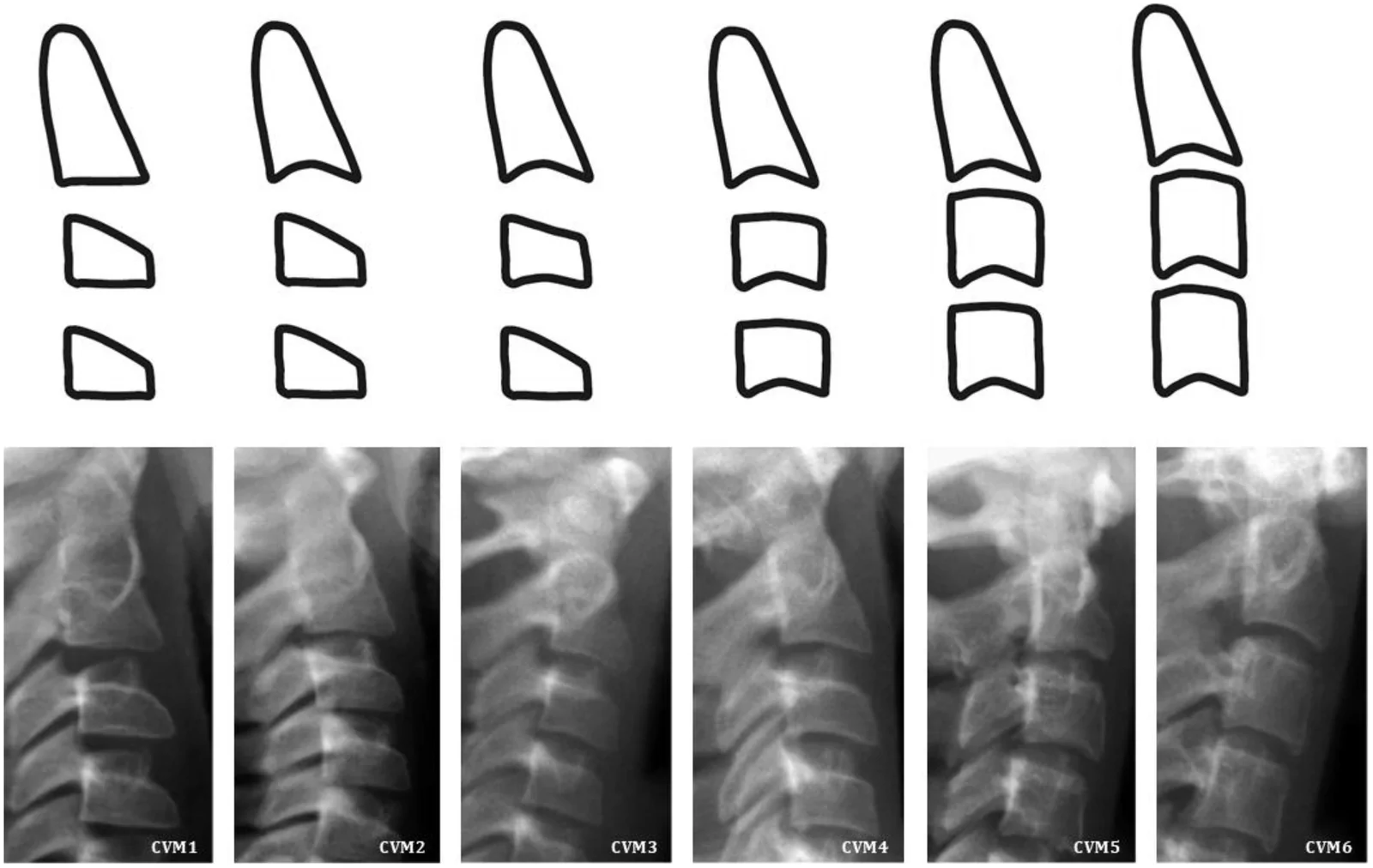
Schematic contours and corresponding lateral cephalometric radiographs illustrating cervical vertebral morphological changes across CVM stages during growth and development. CVM1: Inferior borders of C2, C3, and C4 are flat; C3 and C4 exhibit a trapezoidal shape. CVM2: A concavity appears at the inferior border of C2; other morphological features remain similar to CVM1. CVM3: Concavities are present at the inferior borders of C2 and C3; C3 and C4 transition from a trapezoidal to a horizontally rectangular shape. CVM4: Concavities are evident at the inferior borders of C2–C4; C3 and C4 exhibit a horizontally rectangular shape. CVM5: Concavities persist at the inferior borders of C2–C4; C3 and C4 transition toward a square shape. CVM6: Concavities remain at the inferior borders of C2–C4; C3 and C4 present as horizontally and/or vertically rectangular.

Initial CVM staging of all radiographic images was independently performed by an orthodontist with more than 10 years of clinical experience and routine use of the CVM method. After a washout period of one month, the same orthodontist re-evaluated all images following anonymization and random reordering. Intra-observer agreement between the two assessments was high $\left(\mathrm{Cohe}n^{\prime }s\;\kappa =0.91\right)$. For images with discrepant classifications between the two assessments, a second orthodontist—also with more than 10 years of clinical experience and long-term familiarity with the same classification method—was consulted. If a consensus classification was reached between the two orthodontists, the image was retained in the dataset; otherwise, the image was excluded. All images were labeled and processed according to this standardized protocol.

#### 2.3.2 ROI cropping

Previous studies have demonstrated that vision-based neural network models focusing on the cervical vertebral region can achieve higher predictive performance than models using full lateral cephalometric images. Accordingly, a YOLOv8-based deep learning model (21) was trained to automatically detect and crop the cervical vertebral region for batch extraction of the region of interest (ROI).

Cervical ROIs were manually annotated using a single bounding box encompassing the cervical vertebral region, with C2–C4 required to be fully included. The superior boundary was standardized at the anatomical junction between C1 and C2, whereas the inferior boundary extended to the lowest clearly visible and non-truncated cervical vertebra. Images in which C2–C4 could not be clearly identified or were substantially obscured by truncation or artifacts were excluded from detector development. Bounding boxes were initially annotated by an orthodontist with more than 10 years of clinical experience and independently reviewed by a second orthodontist, with discrepancies resolved by consensus. Final annotations were exported in YOLO format as normalized bounding-box coordinates.

The ROI detector used in the present study was independently developed in a separate detector-development study and was subsequently applied as a fixed upstream tool for automated cervical ROI extraction. The detector-development dataset comprised 950 lateral cephalometric images, which were divided into training (*n* = 665), validation (*n* = 143), and internal test (*n* = 142) sets at a ratio of 70:15:15 while maintaining the proportional representation of the imaging sources. A YOLOv8s model initialized with COCO-pretrained weights was implemented using the Ultralytics framework in Python with PyTorch. Input images were resized to 1,024 pixels, and the model was trained for a maximum of 100 epochs with an initial learning rate of 0.001. Early stopping was applied when validation performance failed to improve for 20 consecutive epochs. Conservative augmentation included rotation up to ±3°, translation up to 2%, scaling up to 10%, and mild shearing; Mosaic augmentation was applied with a probability of 0.5, whereas MixUp and copy-paste augmentation were disabled. Training was performed on an NVIDIA RTX 4080 SUPER GPU. Detector performance was evaluated using precision, recall, and mAP_50–95_. The corresponding detector performance is summarized in Table 2.

**Table 2:** Performance of the YOLOv8-based cervical vertebral ROI detector.

Model	Precision	Recall	mAP_50–95_
YOLOv8s	0.993	0.991	0.865

The trained detector was then applied to the radiographs in the present CVM-classification study for automated cervical ROI extraction. All cropped ROI images were resized to 512 × 512 pixels without geometric distortion, ensuring preservation of anatomical information while maintaining consistency for training and inference across both CNN-based and transformer-based models (22, 23). Representative raw images and corresponding automatically extracted cervical ROIs are shown in Figure 2.

**Figure 2:**
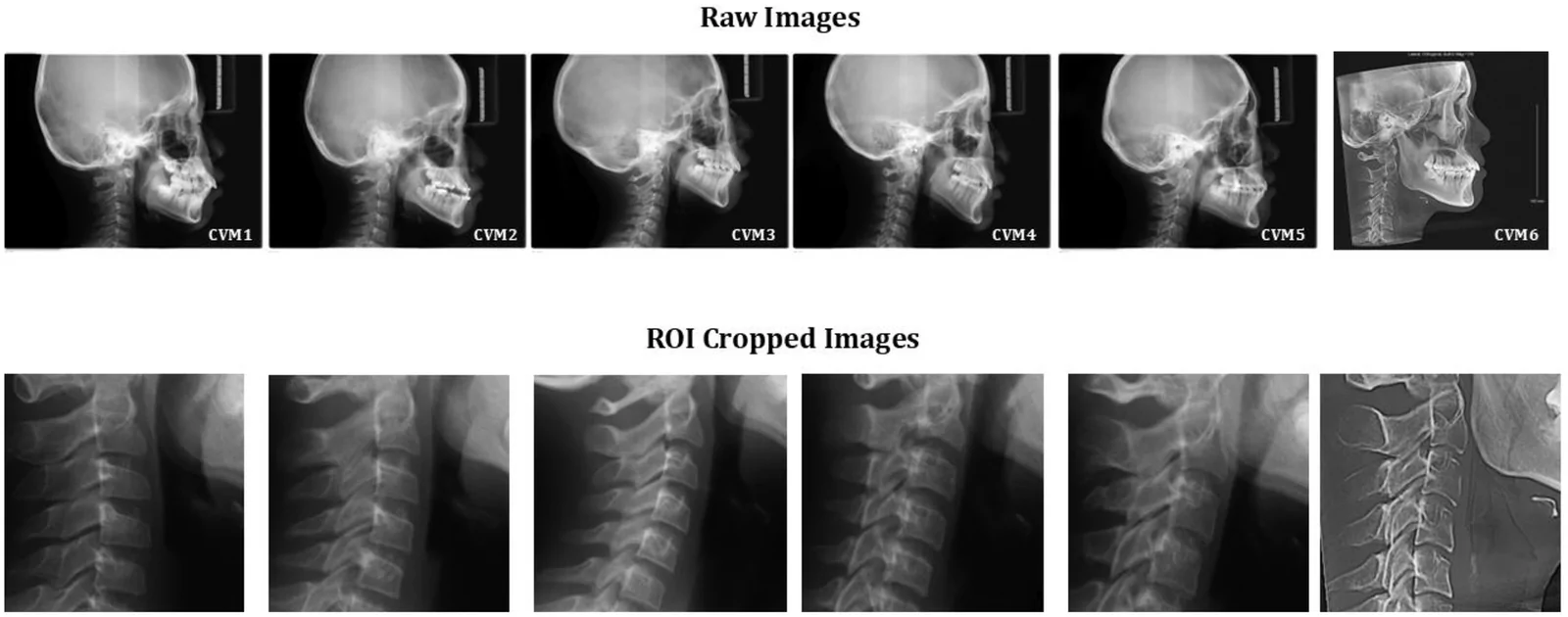
Representative raw lateral cephalometric images and the corresponding cervical vertebral region–of–interest (ROI) images obtained by automated cropping.

### 2.4 Model architecture and implementation

Two deep learning architectures were employed for model training and inference: ResNet-50 and Swin Transformer. ResNet-50 represents a convolutional neural network (CNN)–based architecture that extracts hierarchical local features through stacked convolutional operations (24), whereas Swin Transformer is a vision transformer–based model that captures multi-scale contextual information using shifted window–based self-attention mechanisms (25). The two architectures were evaluated in parallel to compare predictive performance under different radiographic image-processing pipelines.

Both models were initialized with weights pretrained on the ImageNet dataset to provide a common baseline for feature representation learning and to facilitate stable optimization given the limited size of medical imaging datasets. Hyperparameter tuning was conducted exclusively within the internal training and validation sets and was intentionally kept to a minimum to preserve model generalizability and reduce the risk of information leakage. The external validation dataset was used solely for independent performance evaluation and was not involved in model architecture selection, parameter tuning, or training procedures.

Intensity-based augmentations were not applied because all images were grayscale, and excessive contrast or brightness perturbations could distort clinically meaningful morphological cues (26). Data augmentation was applied to the training set only to simulate clinically plausible acquisition variability while preserving diagnostically relevant cervical vertebral morphology (27). Mild geometric augmentation consisted of random in-plane rotation (maximum ±5°) and slight translational perturbations (maximum shift of 2% along both axes). Horizontal flipping, scaling, and shearing were not used to avoid altering anatomical proportions or ordinal CVM morphology. Validation and external testing were performed without any augmentation.

### 2.5 Ablation study and preprocessing analysis

All raw lateral cephalometric images were classified into six cervical vertebral maturation (CVM) stages (CVM1–CVM6) according to the established staging method. The distribution of images across CVM stages is summarized in Table 3 and was consistent with distributions typically observed in clinical populations.

**Table 3:** Baseline characteristics of the development and external validation cohorts.

Characteristic	Development cohort (*n* = 708)	External validation cohort (*n* = 200)
Age, years	8–39	8–36
Sex, female/male	360/348	111/89
CVM1	86	20
CVM2	118	20
CVM3	124	21
CVM4	156	70
CVM5	179	52
CVM6	45	17

Although a moderate class imbalance was observed—particularly between CVM 5 and CVM 6—this distribution reflects the natural prevalence pattern in sequentially collected clinical cases rather than artificial sampling bias. CVM 5 and CVM 6 both represent late skeletal maturation stages with limited mandibular/maxillary growth potential, and in clinical practice these stages are often combined due to minimal therapeutic distinction. Given that the primary objective of this ablation study was to evaluate the influence of preprocessing strategies and architectural paradigms rather than to optimize minority-class sensitivity, explicit imbalance mitigation techniques were not applied.

Raw, unprocessed images were first evaluated using both ResNet-50 and Swin Transformer models to establish baseline predictive performance (P1). For the remaining pipelines, cervical vertebral regions were automatically extracted from the raw images to generate region-of-interest (ROI) inputs for model prediction. The resulting ROI images were directly used as the input for the (P2) pipeline without any further preprocessing. The ROI images were then subjected to a series of preprocessing strategies, including min–max normalization alone (P3), min–max normalization combined with contrast-limited adaptive histogram equalization (CLAHE) (P4), min–max normalization combined with *Z*-score normalization (P5), and min–max normalization combined with histogram-based processing (P6).

To control training-related variables when evaluating the effects of image preprocessing, both architectures were trained using a unified optimization protocol. This controlled design was intended to facilitate comparison of preprocessing effects under consistent training conditions rather than to achieve architecture-specific optimal performance. Accordingly, the resulting differences between ResNet-50 and Swin Transformer should be interpreted within this standardized experimental framework rather than as a comparison of their maximum achievable performance. Detailed training hyperparameters and computational settings are provided in Supplementary File 1.

Each preprocessed dataset was independently evaluated using both model architectures, enabling systematic comparison of predictive performance across different preprocessing pipelines. A summary of all preprocessing strategies and corresponding model configurations is provided in Table 4. Representative examples of raw images, ROI-cropped images, and the resulting appearances after each preprocessing pipeline are illustrated in Figure 3, highlighting the visual effects of different normalization and enhancement strategies on cervical vertebral morphology.

**Table 4:** Summary of image-processing pipelines and model configurations evaluated in this study.

Pipeline ID	Input image type	ROI extraction	Preprocessing strategy	Model architecture
P1	Raw lateral cephalogram	No	None	ResNet-50/Swin Transformer
P2	ROI image (512 × 512)	Yes (YOLOv8)	None	
P3	ROI image (512 × 512)	Yes (YOLOv8)	Min–max normalization	
P4	ROI image (512 × 512)	Yes (YOLOv8)	Min–max + CLAHE	
P5	ROI image (512 × 512)	Yes (YOLOv8)	Min–max + *Z*-score normalization	
P6	ROI image (512 × 512)	Yes (YOLOv8)	Min–max + histogram-based processing	

**Figure 3:**
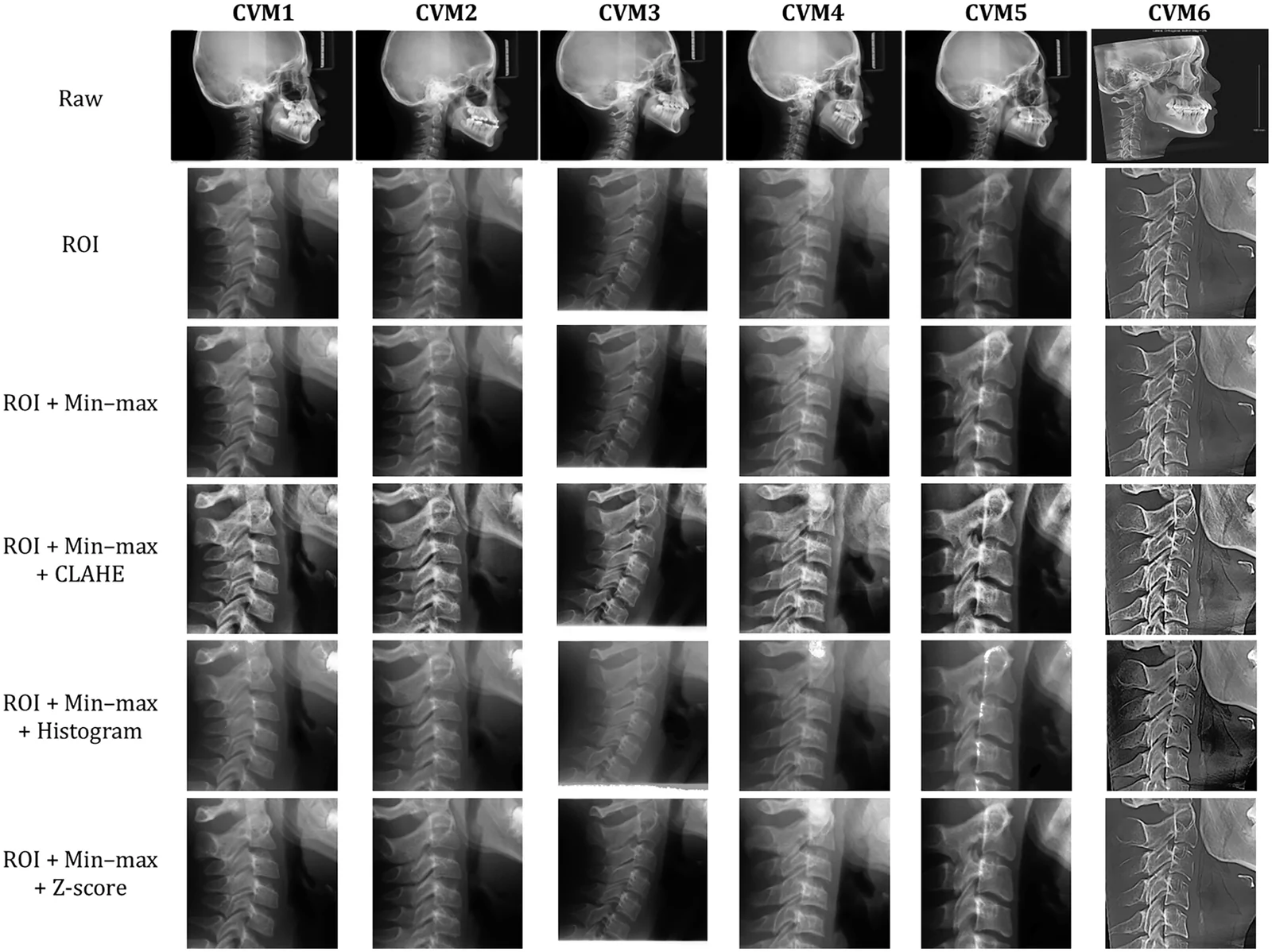
Representative examples of raw lateral cephalometric images, automatically extracted cervical vertebral region-of-interest (ROI) images, and corresponding appearances after different image-processing pipelines.

### 2.6 Evaluation metrics

Model performance was assessed using multiple complementary metrics, including exact-stage accuracy, Macro-F1, confusion matrices, and class-wise precision, recall, and F1-score. Given the unequal distribution of CVM stages, accuracy was not interpreted as a standalone primary measure. Macro-F1 was used as a class-balanced summary metric by assigning equal weight to each stage, while class-wise metrics were used to characterize stage-specific performance.

Given the ordinal nature of CVM staging, adjacent accuracy was defined as the proportion of predictions that either matched the reference label or differed by one adjacent stage (±1 CVM stage). Severe misclassification rate was defined as the proportion of predictions deviating by two or more CVM stages.

For the external validation dataset, 95% confidence intervals (CIs) for exact-stage accuracy were estimated using bootstrap resampling with 1,000 iterations. Because identical external cases were evaluated across preprocessing pipelines and model architectures, pairwise differences in exact-stage accuracy were assessed using paired bootstrap resampling. Two-sided *P* values < 0.05 were considered statistically significant. The external dataset was used exclusively for model evaluation and was not involved in model training or model selection.

All metrics were computed separately for the internal validation dataset and the held-out external test dataset. The external dataset, consisting exclusively of images acquired using the Vatech system, was used solely for model evaluation and was not involved in model training or selection.

### 2.7 Explainable artificial intelligence (XAI) analysis

Post hoc explainable artificial intelligence analyses were performed to explore the image regions contributing to model predictions. For the ResNet-50 model, gradient-weighted class activation mapping (Grad-CAM) was applied to generate class-discriminative saliency maps based on the final convolutional layer. Heatmaps were overlaid on the corresponding input images for visualization.

For the Swin Transformer model, attention rollout was employed to visualize aggregated self-attention maps across transformer layers. Attention maps were projected back into the image space to identify regions receiving higher attention during inference.

XAI analyses were conducted on both correctly classified and misclassified samples from the validation and external test sets. These visualizations were used to qualitatively assess whether model attention aligned with anatomically relevant cervical vertebral structures, rather than serving as quantitative performance metrics.

### 2.8 Overall experimental framework

To provide a structured overview of the proposed framework, a schematic illustration of the complete workflow is presented in Figure 4. Detailed architectural components of the ResNet-50 and Swin Transformer models are summarized in Figure 5.

**Figure 4:**
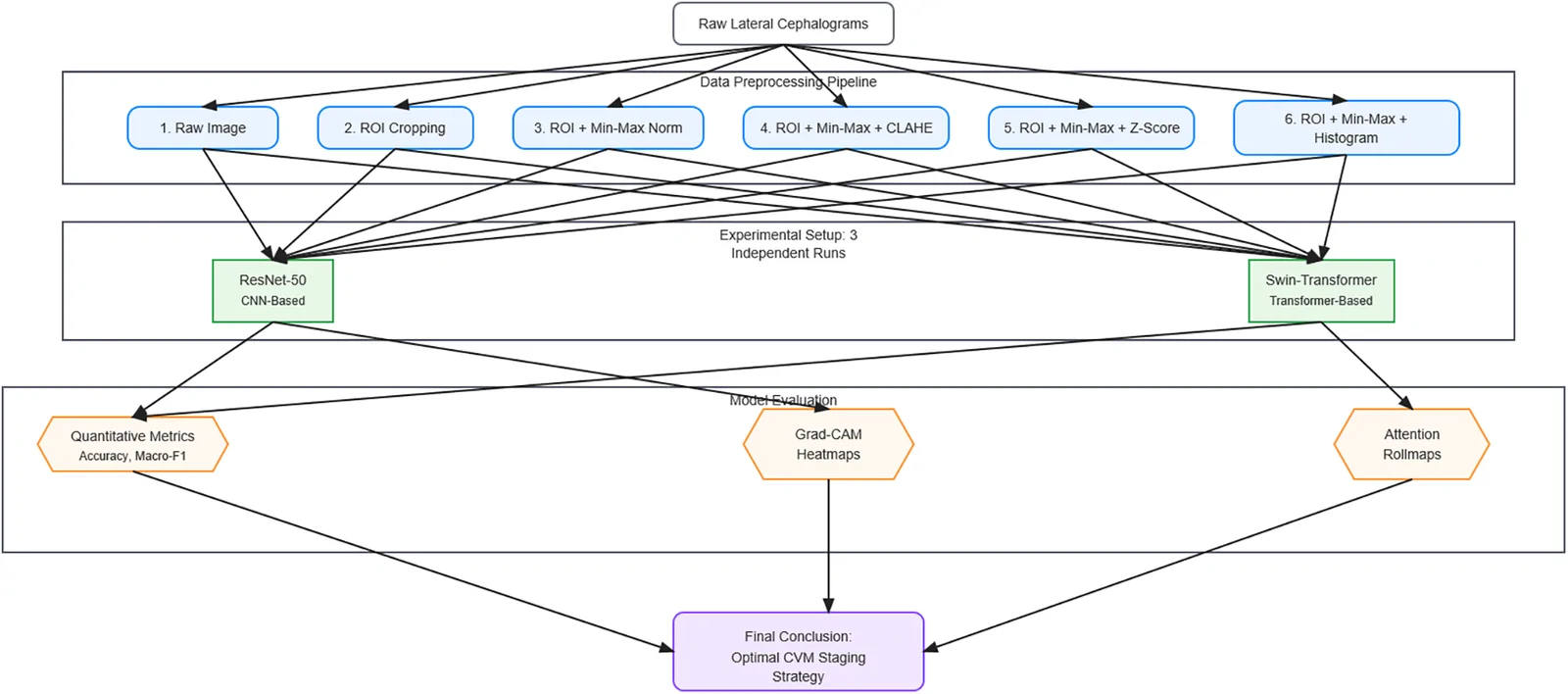
Overall experimental framework for CVM staging. Lateral cephalograms were processed using multiple preprocessing strategies and subsequently analyzed using two independent deep learning architectures (ResNet-50 and Swin Transformer). Model performance was assessed through quantitative metrics and interpretability visualization, including Grad-CAM and attention maps.

**Figure 5:**
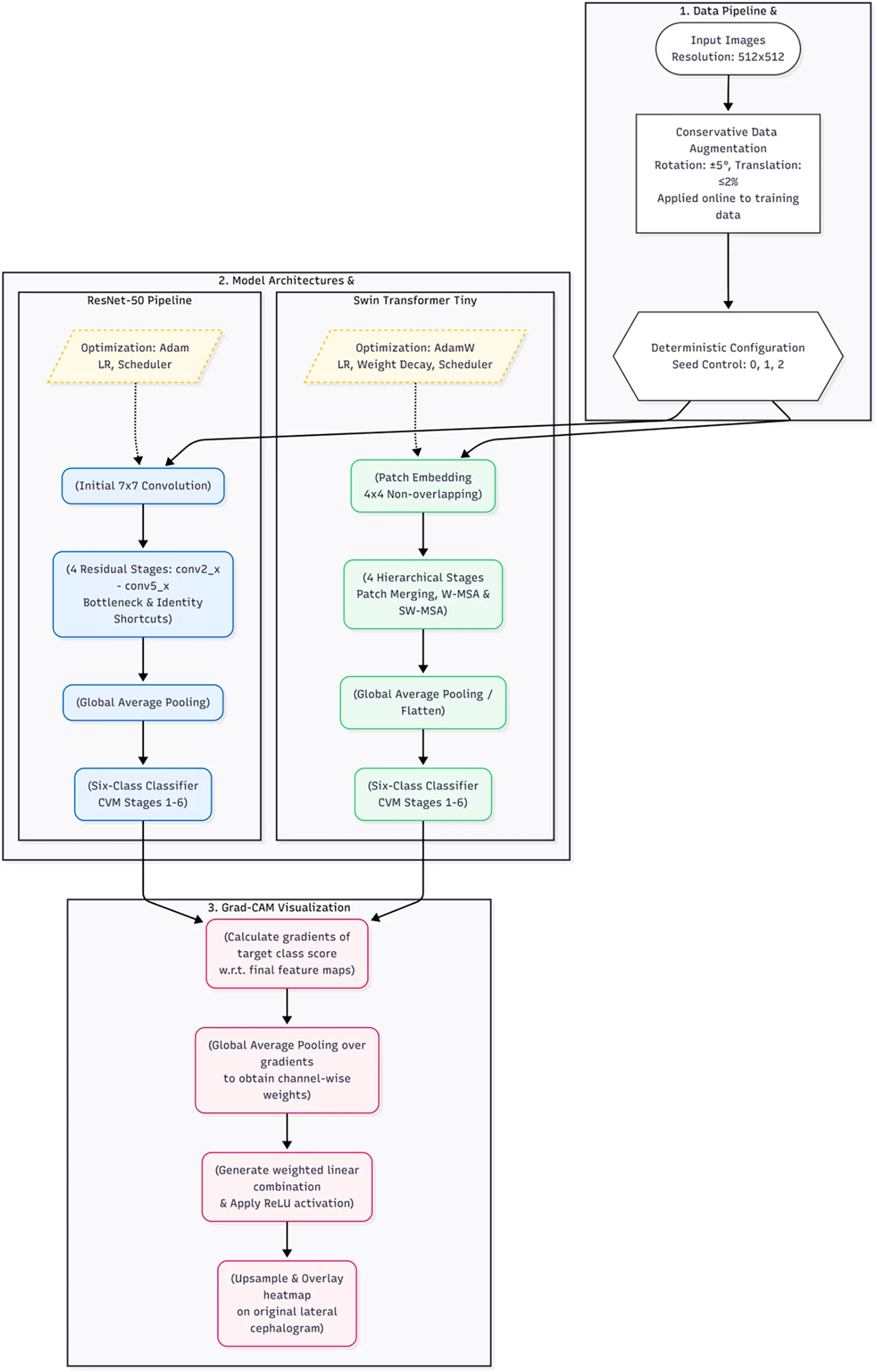
Architecture and interpretability workflow of the proposed CVM staging framework. Input cephalograms (512 × 512) underwent conservative online geometric augmentation (±5° rotation, ≤2% translation) under deterministic configuration. The ResNet-50 pipeline consists of an initial 7 × 7 convolution followed by four residual stages and a global average pooling classifier. The Swin Transformer Tiny architecture employs 4 × 4 patch embedding, hierarchical shifted-window self-attention blocks, and patch merging layers. Grad-CAM generates class-discriminative activation maps via gradient-based weighting of final feature maps.

All models were implemented in PyTorch (version 2.5.1) and trained on an NVIDIA RTX 4080 SUPER GPU using CUDA 12.1. Experiment configuration, data handling, and training workflow management were conducted within a unified experimental framework, with consistent settings applied across all preprocessing conditions to ensure fair and reproducible model comparison.

## 3 Results

### 3.1 Dataset characteristics

A total of 708 lateral cephalometric radiographs acquired using Sirona, NewTom, and Meyer systems were used for model training and internal validation, while 200 images from the Vatech system were reserved exclusively as an independent external validation dataset. The internal dataset was stratified into training and validation subsets at an 80:20 ratio to ensure a balanced distribution of CVM stages. The detailed composition of the dataset by imaging vendor and data split is presented in Table 5.

**Table 5:** Dataset composition across vendors and data splits.

Vendor	Train (n)	Validation (n)	External test (*n*)	Total
Sirona	352	88	—	440
NewTom	120	30	—	150
Meyer	94	24	—	118
Vatech	—	—	200	200
Total	566	142	200	908

### 3.2 Classification performance across pipelines and architectures

Internal validation accuracy varied within a narrow range across preprocessing pipelines for both ResNet-50 and Swin Transformer models, indicating limited sensitivity to image-level processing strategies during internal testing (Table 6 and Figure 6). Although specific configurations yielded marginally higher internal accuracy, these differences were inconsistent across architectures.

**Table 6:** Internal validation and external test accuracy of resNet-50 and swin transformer models across different image-processing pipelines with different random seeds (0, 1, and 2).

Image-processing pipeline	ResNet-50 (Internal)	ResNet-50 (External)	Swin transformer (Internal)	Swin transformer (External)
P1	0.53 ± 0.04	0.43 ± 0.05	0.49 ± 0.03	0.36 ± 0.02
P2	0.53 ± 0.03	0.37 ± 0.02	0.49 ± 0.03	0.36 ± 0.01
P3	0.53 ± 0.02	0.46 ± 0.03	0.52 ± 0.04	0.34 ± 0.02
P4	0.54 ± 0.03	0.43 ± 0.04	0.63 ± 0.02	0.42 ± 0.02
P5	0.52 ± 0.04	0.47 ± 0.02	0.56 ± 0.02	0.29 ± 0.02
P6	0.49 ± 0.03	0.40 ± 0.03	0.56 ± 0.03	0.35 ± 0.01

**Figure 6:**
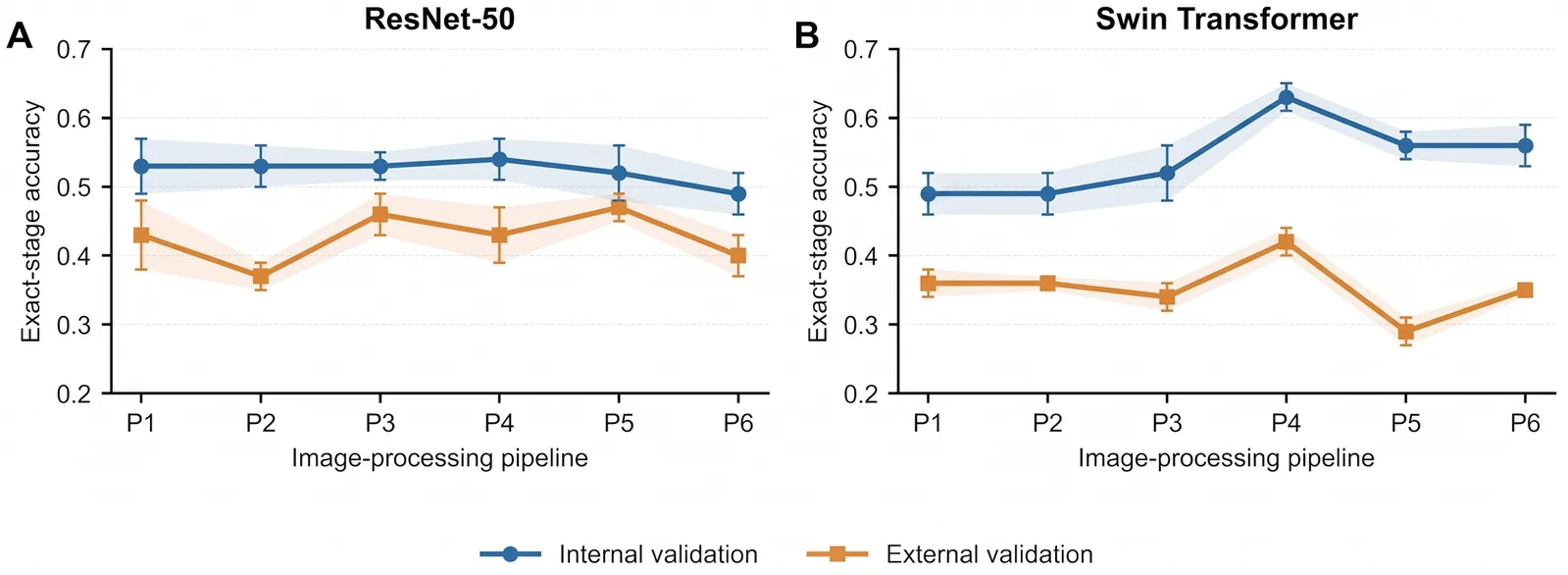
Classification accuracy across image-processing pipelines for **(A)** ResNet-50 and **(B)** Swin Transformer models.

Crucially, these variations did not translate into stable improvements on the external test dataset. External accuracy remained uniformly lower than internal validation accuracy across all pipelines. Pipelines showing relatively higher internal performance did not reliably generalize to heterogeneous imaging sources. These findings indicate that incremental image-level preprocessing does not substantially alter the generalization behavior of deep learning models for CVM staging on 2D lateral cephalograms.

### 3.3 External validation and error pattern analysis

On the independent external dataset, both models showed moderate exact-stage classification performance across preprocessing pipelines (Figure 7). For ResNet-50, external exact-stage accuracy ranged from 0.37 to 0.47, with the highest value observed for P5 (0.47, 95% CI: 0.40–0.54). For the Swin Transformer, external exact-stage accuracy ranged from 0.29 to 0.42, with the highest value observed for P4 (0.42, 95% CI: 0.35–0.49). Corresponding Macro-F1 values ranged from 0.261 to 0.362 for ResNet-50 and from 0.189 to 0.300 for the Swin Transformer. Pipeline-specific Macro-F1 values for all model–pipeline combinations are provided in Supplementary File 2.

**Figure 7:**
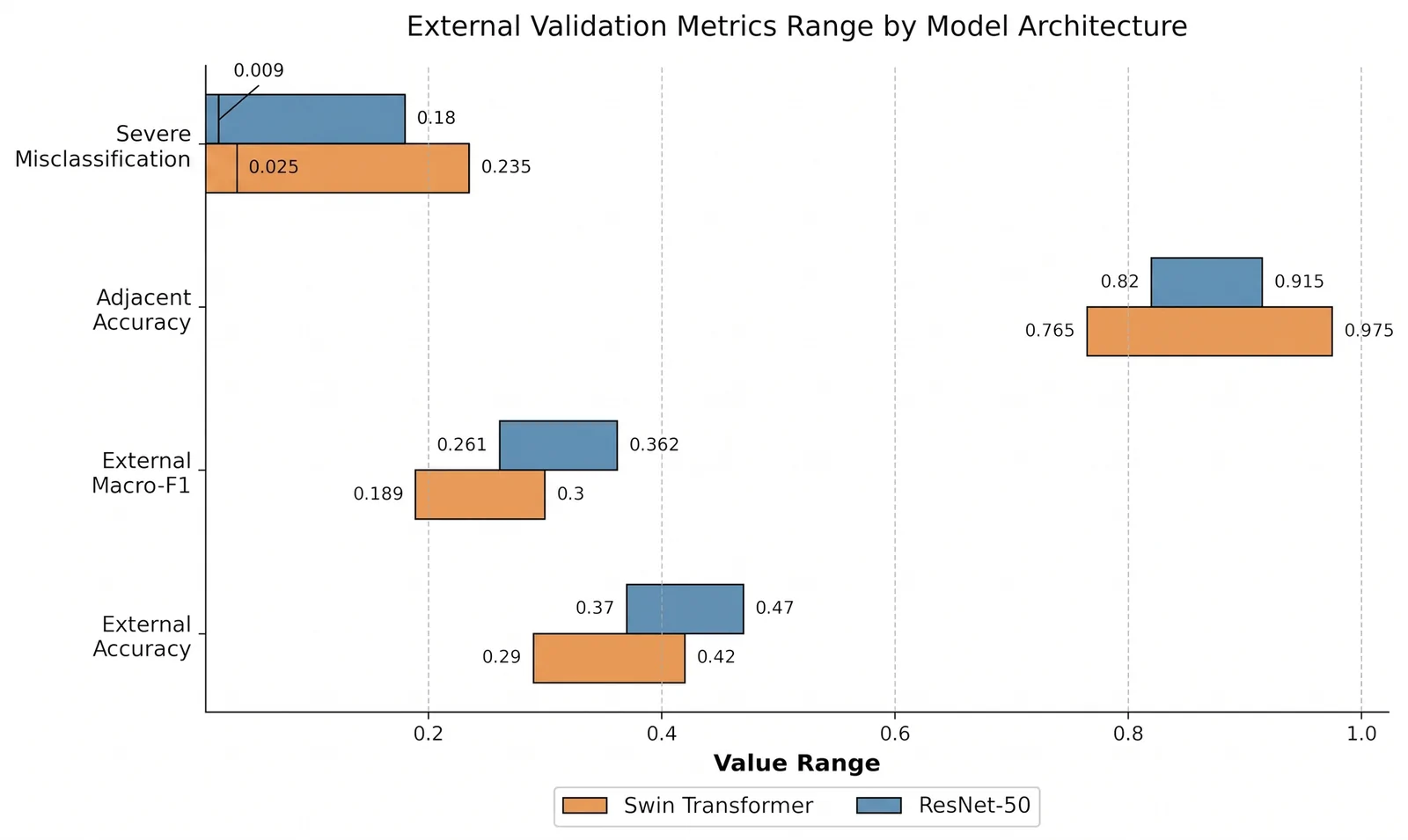
External validation metric ranges across preprocessing pipelines for resNet-50 and swin transformer models. The horizontal bars represent the minimum-to-maximum values obtained across preprocessing pipelines P1–P6. For external exact-stage accuracy, the best- and worst-performing pipelines were P5 and P2 for ResNet-50, and P4 and P5 for the Swin Transformer, respectively. Corresponding ranges of external Macro-F1, adjacent-stage accuracy, and severe misclassification rate are also shown.

Adjacent-stage accuracy remained substantially higher than exact-stage accuracy, ranging from 0.820 to 0.915 for ResNet-50 and from 0.765 to 0.975 for the Swin Transformer, whereas severe misclassification rates ranged from 0.085 to 0.180 and from 0.025 to 0.235, respectively.

Paired bootstrap comparisons (1,000 resamples) showed that, for ResNet-50, the higher exact-stage accuracy of P5 reached statistical significance only when compared with P2 (*P* = 0.032), whereas differences between P5 and P1 (*P* = 0.124), P3 (*P* = 0.286), P4 (*P* = 0.410), and P6 (*P* = 0.185) were not statistically significant. Similarly, for the Swin Transformer, no statistically significant differences in exact-stage accuracy were observed between the numerically best-performing P4 pipeline and P1 (*P* = 0.215), P2 (*P* = 0.084), P3 (*P* = 0.342), P5 (*P* = 0.478), or P6 (*P* = 0.160).

These findings indicate that the numerically best-performing preprocessing pipeline should not necessarily be interpreted as statistically superior to alternative pipelines. In addition, higher adjacent-stage agreement did not necessarily eliminate clinically relevant severe errors. Class-wise precision, recall, and F1-score for the best-performing ResNet-50 and Swin Transformer pipelines on the external validation dataset are provided in Supplementary File 2.

### 3.4 Qualitative interpretability analysis

Explainable AI visualizations (Grad-CAM for ResNet-50 and attention rollout for Swin Transformer) revealed distinct model behaviors (Figures 8, 9). For predictions based on raw images, ResNet-50 frequently focused on regions outside the target C2–C4 vertebral area, such as the cranial base, dentition, and the spinous process of the first cervical vertebra. In contrast, the Swin Transformer distributed attention across the entire cervical region and posterior cranial area. Notably, non-anatomical artifacts—including image scale markers (rulers) and peripheral light bands—were frequently highlighted in attention maps for both correctly and incorrectly classified cases. These observations suggested potential shortcut learning patterns.

**Figure 8:**
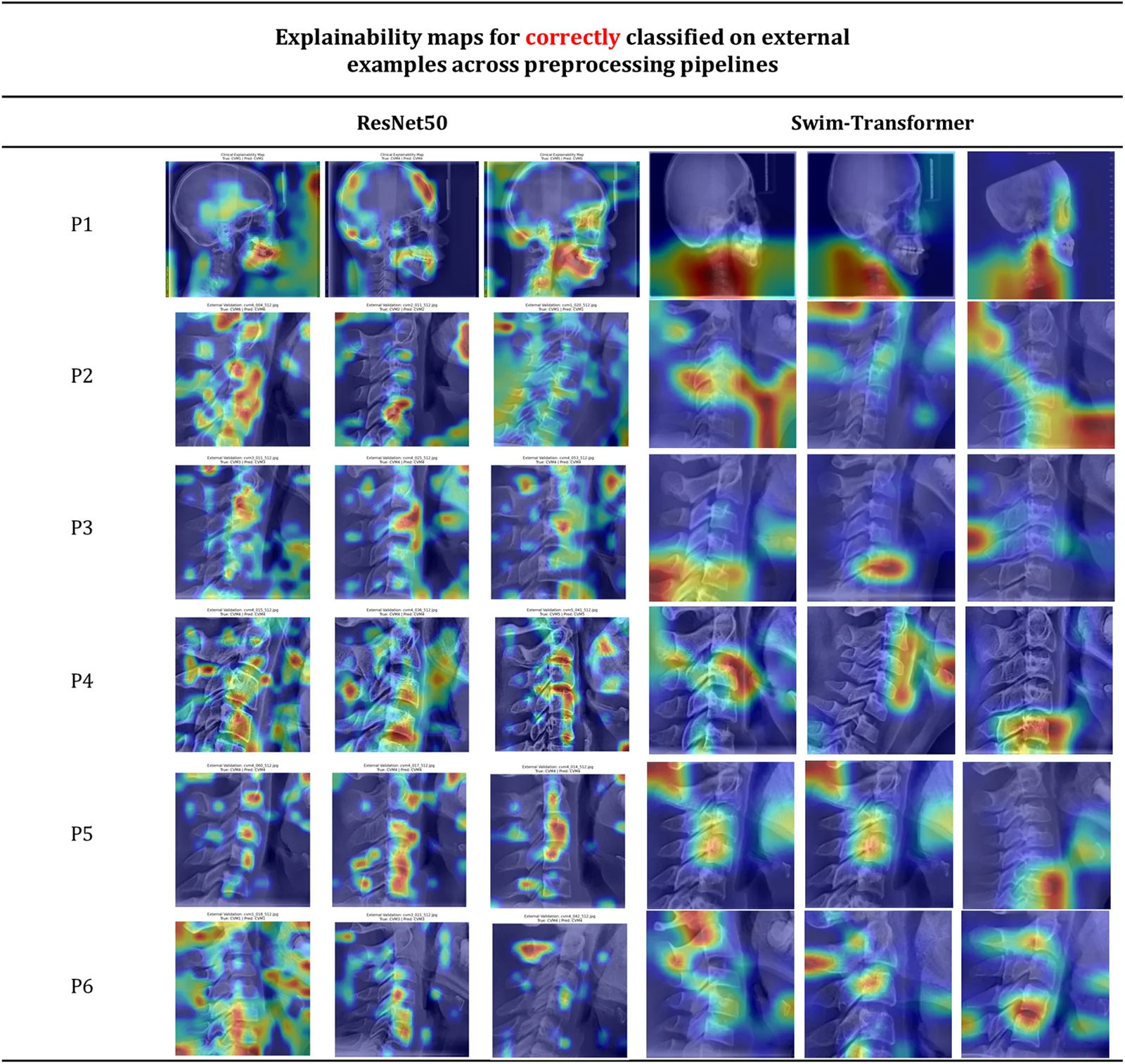
Explainability maps for representative correctly classified cases from the external validation dataset across preprocessing pipelines P1–P6. Grad-CAM visualizations from the ResNet-50 model (left three columns) and attention rollout maps from the Swin Transformer model (right three columns) are shown.

**Figure 9:**
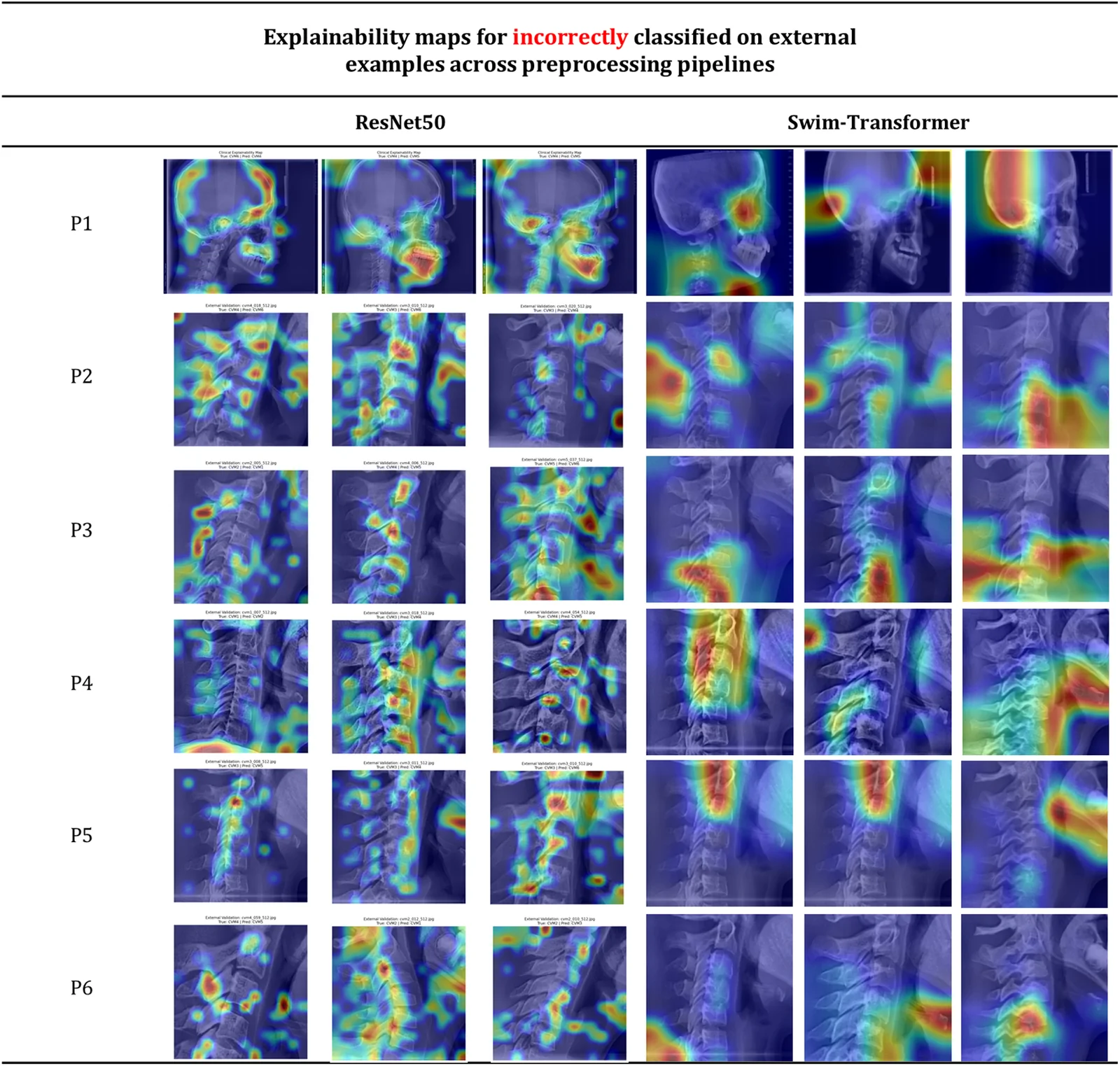
Explainability maps for representative misclassified cases from the external validation dataset across preprocessing pipelines P1–P6. Grad-CAM visualizations from the ResNet-50 model (left three columns) and attention rollout maps from the Swin Transformer model (right three columns) are shown.

### 3.5 Impact of preprocessing on model attention

No single preprocessing pipeline consistently outperformed the others across both architectures. Based on external exact-stage accuracy, P5, consisting of ROI + min–max + *Z*-score normalization, achieved the best performance for ResNet-50, whereas P4, consisting of ROI + min–max + CLAHE, achieved the best performance for the Swin Transformer. This indicates that the effectiveness of preprocessing was architecture-dependent rather than universally transferable across model types. Inconsistencies were further highlighted by identical images yielding different predictions under different preprocessing pipelines; for instance, subtle feature enhancement strategies occasionally shifted predictions from correct to incorrect stages, or vice versa, without a consistent anatomical basis.

## 4 Discussion

Previous studies have extensively explored deep learning–based approaches for cervical vertebral maturation (CVM) assessment (28–30), yet reported predictive performance has varied widely. This variability can be attributed, in part, to heterogeneity in model architectures, hyperparameter optimization strategies, and image-preprocessing pipelines, which collectively limit reproducibility and complicate direct comparison across studies (16, 31, 32). Additionally, concerns regarding data privacy and the “black-box” nature of many models often offer little transparency into how predictions are generated, suggesting that reported results should be interpreted with caution (33–35). Despite increasing reports of high internal accuracy, a persistent gap remains between experimental performance and routine clinical adoption (36). The present study aimed to address this gap by emphasizing methodological transparency, robustness, and clinical relevance.

In this work, we systematically compared a classical convolutional neural network (CNN) architecture, ResNet-50, with a transformer-based architecture, the Swin Transformer. ResNet-50 relies on local receptive fields to capture fine-grained morphological characteristics such as vertebral curvature (24, 26), whereas the Swin Transformer employs shifted window–based self-attention to model broader contextual relationships (25, 37). A central objective was to assess whether specific image-processing pipelines exert consistent effects across these architectures. Our findings indicate that preprocessing strategies differentially influence CNN- and transformer-based models, underscoring the need for architecture-aware evaluation rather than assuming universal benefits of a single pipeline. Beyond quantitative metrics, we emphasized interpretability using Grad-CAM and attention rollout to examine whether predictive performance aligned with established CVM criteria, such as inferior border concavities and shape transitions described by Baccetti and colleagues.

The architecture-dependent numerical performance pattern may be partly related to the different feature-extraction mechanisms of the two models. For ResNet-50, P5 yielded the highest external point estimate, whereas P4 yielded the highest external point estimate for the Swin Transformer. However, statistical comparisons indicated that several differences between preprocessing pipelines were not significant, and these numerical rankings should therefore be interpreted cautiously rather than as evidence of universal pipeline superiority. *Z*-score normalization may reduce vendor-related grayscale variability for CNN-based feature extraction, whereas CLAHE may enhance local contrast around vertebral borders and concavities relevant to transformer-based representations; however, these mechanistic interpretations remain exploratory. This may have improved the visibility of spatially distributed morphological cues captured by self-attention mechanisms. Therefore, the present findings do not support a universally optimal preprocessing pipeline but instead highlight the importance of selecting preprocessing strategies according to the underlying model architecture.

A strictly defined manual CVM staging protocol was adopted to establish a reliable reference standard, as label quality directly constrains the upper bound of achievable performance. In supervised learning, annotation inaccuracies introduce label noise, which can distort learned feature–label relationships. For example, if a radiograph exhibiting late-stage maturation is mislabeled as an early stage, the model may associate advanced morphological characteristics with earlier growth phases. To mitigate this, we prioritized intra-observer consistency by using a single experienced orthodontist for two independent sessions separated by a one-month washout period. Although multi-annotator designs are common, CVM assessment is inherently subjective because skeletal maturation represents a continuous biological process rather than discrete transitions. As noted by McNamara, “A person does not go to bed in the evening at CS2 and wake up the next morning at CS3” (2). By prioritizing intra-observer consistency and utilizing a secondary senior review for discrepant cases, we aimed to preserve the continuity of morphological feature representation, facilitating stable learning of gradual shape transitions. Future orthodontic AI studies may similarly benefit from balancing clinical consensus with high-calibration primary annotation to reduce label noise in ordinal classification tasks.

Our experiments revealed a substantial gap between training and validation performance. Although training accuracy approached unity, internal-validation accuracy ranged from only 0.49 to 0.63, indicating considerable overfitting under the present training settings. Performance generally decreased further on the held-out vendor dataset, suggesting that cross-vendor distribution differences represented an additional generalization challenge beyond within-development-set overfitting. The gradual and potentially ambiguous morphological transitions between adjacent CVM stages may further contribute to the difficulty of generalization. These findings indicate that high training performance should not be interpreted as evidence of robust CVM classification and highlight the importance of both internal validation and independent held-out evaluation when assessing model performance. Clinically, misclassification between adjacent stages is less likely to alter treatment timing than errors spanning multiple stages, suggesting that optimized preprocessing may reduce clinically consequential errors even when exact-stage accuracy is limited.

Importantly, the goal of this study was not to maximize CVM classification accuracy through increasingly specialized prediction strategies, but to examine whether predictive performance was accompanied by anatomically plausible model attention. Recent studies have shown that CVM assessment can be formulated in substantially different ways, including hierarchical multi-stage classification (38), explicit landmark- and morphology-based analysis (39), integration of anatomical landmarks with clinical metadata (40), and machine-learning approaches developed using highly consistent consensus-based reference labels (41). Collectively, these studies suggest that the representation of vertebral maturation, the formulation of the prediction task, and the quality of reference labels may all influence model performance. The present study addresses a complementary question. By retaining a conventional six-stage image-classification framework under standardized training conditions, we examined whether predictive performance was accompanied by attention to the anatomical structures conventionally used for CVM assessment and whether these patterns were maintained under held-out vendor evaluation.

Crucially, XAI analyses revealed that predictions were frequently influenced by features outside the canonical C2–C4 region, including the hyoid bone, airway morphology, and dental development. This pattern is consistent with shortcut learning, or the “Clever Hans” effect, in which models exploit correlated auxiliary cues rather than intended anatomical features (42, 43). Although such cues may be biologically associated with maturation, reliance on them introduces vulnerability in patients with atypical growth patterns or prior orthodontic intervention. Models also demonstrated sensitivity to non-anatomical confounders such as thyroid shields, orthodontic appliances and image scale markers. While ROI extraction mitigated these confounders, the modest performance reduction in some ROI-based experiments suggests that global contextual cues may contribute to predictions. Furthermore, feature visualizations indicated that models could learn vendor-specific signatures related to acquisition parameters (43), highlighting the importance of intensity normalization for cross-vendor generalizability (44).

Although AI has advanced medical image analysis, translation into routine orthodontic practice requires caution. CVM assessment directly informs treatment timing, and errors may have irreversible consequences. Accordingly, increasing availability of AI-based CVM tools necessitates heightened clinical vigilance rather than uncritical adoption. From an ethical perspective, perceived algorithmic “objectivity” may create a risk of algorithmic authority, whereby AI outputs overshadow nuanced clinical judgment. Experienced orthodontists integrate multiple growth indicators beyond radiographic morphology, including growth velocity, sexual maturation, and dentofacial development. A model relying on a single two-dimensional image cannot account for this broader context. Incorrect AI recommendations may therefore result in missed optimal treatment windows. Within the models and experimental conditions evaluated in this study, predictive performance was not always accompanied by attention patterns consistent with the anatomical criteria conventionally used for CVM assessment. This finding suggests that classification performance alone may provide an incomplete characterization of model behavior, particularly under cross-vendor distribution shift. Attention to anatomical or imaging-related information beyond the canonical C2–C4 criteria may reflect alternative predictive cues, the clinical and biological significance of which requires further investigation.

Several limitations should be acknowledged. Although high intra-observer agreement was observed $\left(\mathrm{Cohe}n^{\prime }s\;\kappa =0.91\right)$, the second orthodontist evaluated only cases with discrepant classifications rather than independently assessing the entire dataset. Therefore, inter-observer agreement across the full dataset could not be quantified, and future studies should incorporate independent assessments by multiple experts to further evaluate the reproducibility of the reference labels. While XAI techniques provided insight into model attention, definitive qualitative interpretation remains challenging; granular attribution methods could bridge this gap. Additionally, owing to dataset limitations, multimodal integration was not explored. Transformer-based models offer opportunities for integrating radiographic data with clinical variables such as chronological age and sex, which future work should investigate to improve robustness and clinical alignment.

Although the present study employed a multi-vendor development dataset, external validation was performed using a single held-out imaging vendor. Therefore, the observed cross-vendor performance should not be interpreted as evidence of generalizability across all imaging systems or clinical centers. Future studies incorporating multiple independent external centers and vendors are needed to more comprehensively assess model generalizability. In addition, the unified optimization protocol was designed to control training-related variables rather than to optimize each architecture independently. Architecture-specific optimization of learning rates, scheduling strategies, and other hyperparameters may improve the absolute performance of either model and potentially modify the relative differences observed between architectures and preprocessing strategies. Furthermore, the present models were trained using a conventional six-class classification framework and did not explicitly encode the ordinal relationships among CVM stages. Although ordinal information was partially incorporated into evaluation through adjacent-stage accuracy and severe misclassification rate, future prediction-oriented studies may further investigate ordinal classification or order-aware learning strategies. Such approaches would complement, rather than replace, the interpretability-focused objective of the present study.

In summary, this study systematically evaluated CVM prediction using ResNet-50 and Swin Transformer models across different image-processing pipelines, followed by evaluation on a held-out imaging vendor and architecture-specific XAI analyses. Under the experimental conditions examined, both models showed lower performance on the held-out vendor dataset than on the internal validation dataset, while preprocessing influenced predictive performance and attention patterns differently across architectures. XAI analyses further showed that model attention was not always restricted to the canonical C2–C4 morphological features used in conventional CVM assessment. These findings should not be generalized to deep learning–based CVM staging as a whole; rather, they highlight the importance of evaluating both predictive performance and model attention when assessing the behavior of specific models under cross-vendor imaging conditions. Further studies involving additional architectures, independent centers, and imaging vendors are needed to determine the generalizability and clinical significance of these observations.

## 5 Conclusion

In the models evaluated in this study, predictive performance and anatomically relevant attention were not always aligned under cross-vendor imaging conditions. These findings suggest that performance metrics alone may not fully characterize model behavior in CVM staging. Therefore, machine learning–based CVM predictions should be interpreted with caution and should not replace clinical judgment.

## Data Availability

The raw data supporting the conclusions of this article will be made available by the authors, without undue reservation.

## References

[1] Franchi L Baccetti T McNamara JA. Mandibular growth as related to cervical vertebral maturation and body height. Am J Orthod Dentofacial Orthop. (2000) 118:335–40. 10.1067/mod.2000.107009 10982936

[2] McNamara JA Franchi L. The cervical vertebral maturation method: a user’s guide. Angle Orthod. (2018) 88:133–43. 10.2319/111517-787.1 29337631 PMC8312535

[3] Lamparski DG. Skeletal age assessment utilizing cervical vertebrae (Master’s thesis). Department of Orthodontics, The University of Pittsburgh, Pittsburgh, Penn (1972).

[4] Hassel B Farman AG. Skeletal maturation evaluation using cervical vertebrae. Am J Orthod Dentofacial Orthop. (1995) 107:58–66. 10.1016/s0889-5406(95)70157-5 7817962

[5] Baccetti T Franchi L McNamara JA. The cervical vertebral maturation (CVM) method for the assessment of optimal treatment timing in dentofacial orthopedics. Semin Orthod. (2005) 11:119–29. 10.1053/j.sodo.2005.04.005

[6] Perinetti G Bianchet A Franchi L Contardo L. Cervical vertebral maturation: an objective and transparent code staging system applied to a 6-year longitudinal investigation. Am J Orthod Dentofacial Orthop. (2017) 151:898–906. 10.1016/j.ajodo.2016.09.026 28457267

[7] O’Reilly MT Yanniello GJ. Mandibular growth changes and maturation of cervical vertebrae–a longitudinal cephalometric study. Angle Orthod. (1988) 58:179–84. 10.1043/0003-3219(1988)058<0179:MGCAMO>2.0.CO;2 10.1043/0003-3219(1988)058<0179:MGCAMO>2.0.CO;2 3164596

[8] Hamidaddin MA. Optimal treatment timing in orthodontics: a scoping review. Eur J Dent. (2024) 18:86–96. 10.1055/s-0043-1768974 37311555 PMC10959601

[9] Perinetti G Primožič J Franchi L Contardo L. Treatment effects of removable functional appliances in pre-pubertal and pubertal class II patients: a systematic review and meta-analysis of controlled studies. PLoS One. (2015) 10:e0141198. 10.1371/journal.pone.0141198 26510187 PMC4624952

[10] Pavoni C Lombardo EC Lione R Faltin K McNamara JA Cozza P. Treatment timing for functional jaw orthopaedics followed by fixed appliances: a controlled long-term study. Eur J Orthod. (2018) 40:430–6. 10.1093/ejo/cjx078 29099926

[11] Franchi L Pavoni C Faltin K McNamara JA Cozza P. Long-term skeletal and dental effects and treatment timing for functional appliances in class II malocclusion. Angle Orthod. (2013) 83:334–40. 10.2319/052912-450.1 22931200 PMC8793654

[12] Baccetti T Franchi L Toth LR McNamara JA. Treatment timing for twin-block therapy. Am J Orthod Dentofacial Orthop. (2000) 118:159–70. 10.1067/mod.2000.105571 10935956

[13] Polizzi A Leonardi R. Automatic cephalometric landmark identification with artificial intelligence: an umbrella review of systematic reviews. J Dent. (2024) 146:105056. 10.1016/j.jdent.2024.105056 38729291

[14] LeCun Y Bengio Y Hinton G. Deep learning. Nature. (2015) 521:436–44. 10.1038/nature14539 26017442

[15] Dosovitskiy A Beyer L Kolesnikov A Weissenborn D Zhai X Unterthiner T. An image is worth 16 × 16 words: Transformers for image recognition at scale. *arXiv [Preprint]*. (2021). 10.48550/arXiv.2010.11929

[16] Kazimierczak W Jedliński M Issa J Kazimierczak N Janiszewska-Olszowska J Dyszkiewicz-Konwińska M. Accuracy of artificial intelligence for cervical vertebral maturation assessment-a systematic review. J Clin Med. (2024) 13:4047. 10.3390/jcm13144047 39064087 PMC11277636

[17] Rouzrokh P Khosravi B Faghani S Moassefi M Vera Garcia DV Singh Y. Mitigating bias in radiology machine learning: 1. Data handling. Radiol Artif Intell. (2022) 4:e210290. 10.1148/ryai.210290 36204544 PMC9533091

[18] Zhang L Wang X Yang D Sanford T Harmon S Turkbey B. Generalizing deep learning for medical image segmentation to unseen domains via deep stacked transformation. IEEE Trans Med Imaging. (2020) 39:2531–40. 10.1109/TMI.2020.2973595 32070947 PMC7393676

[19] Purwono P Wulandari ANE Nisa K. Explainable artificial intelligence (XAI) in medical imaging: techniques, applications, challenges, and future directions. Adv Mech Mechatron Syst. (2025) 1:52–66. 10.53623/amms.v1i1.692

[20] Mongan J Moy L Kahn CE. Checklist for artificial intelligence in medical imaging (CLAIM): a guide for authors and reviewers. Radiol Artif Intell. (2020) 2:e200029. 10.1148/ryai.2020200029 33937821 PMC8017414

[21] Jocher G Qiu J Chaurasia A. Ultralytics YOLO. (2023). Available online at: https://github.com/ultralytics/ultralytics (Accessed January 6, 2026).

[22] Sabottke CF Spieler BM. The effect of image resolution on deep learning in radiography. Radiol Artif Intell. (2020) 2:e190015. 10.1148/ryai.2019190015 33937810 PMC8017385

[23] Atici SF Ansari R Allareddy V Suhaym O Cetin AE Elnagar MH. Aggregatenet: a deep learning model for automated classification of cervical vertebrae maturation stages. Orthod Craniofac Res. (2023) 26(Suppl 1):111–7. 10.1111/ocr.12644 36855827

[24] He K Zhang X Ren S Sun J. Deep residual learning for image recognition. *arXiv [Preprint]*. (2015). 10.48550/arXiv.1512.03385

[25] Liu Z Lin Y Cao Y Hu H Wei Y Zhang Z. Swin transformer: hierarchical vision transformer using shifted windows. *arXiv [Preprint]*. (2021). 10.48550/arXiv.2103.14030

[26] Seo H Hwang J Jeong T Shin J. Comparison of deep learning models for cervical vertebral maturation stage classification on lateral cephalometric radiographs. J Clin Med. (2021) 10:3591. 10.3390/jcm10163591 34441887 PMC8397111

[27] Goceri E. Medical image data augmentation: techniques, comparisons and interpretations. Artif Intell Rev. (2023) 56:12561–605. 10.1007/s10462-023-10453-z PMC10027281 37362888

[28] Kavousinejad S Ebadifar A Tehranchi A Zakermashhadi F Dalaie K. Determination of cervical vertebral maturation using machine learning in lateral cephalograms. J Dent Res Dent Clin Dent Prospects. (2024) 18:232–41. 10.34172/joddd.41114 39895683 PMC11786010

[29] Li H Chen Y Wang Q Gong X Lei Y Tian J. Convolutional neural network-based automatic cervical vertebral maturation classification method. Dentomaxillofac Radiol. (2022) 51:20220070. 10.1259/dmfr.20220070 35612567 PMC10043621

[30] Jiang F, Abdulqader AA, Yan Y, Cheng F, Xiang T, Yu J, et al. Deep learning based quantitative cervical vertebral maturation analysis. *Head Face Med.* (2025) 21:20. doi: 10.1186/s13005-025-00498-6 10.1186/s13005-025-00498-6 PMC11938625 40140932

[31] Sadeghi TS Ourang SA Sohrabniya F Sadr S Shobeiri P Motamedian SR. Performance of artificial intelligence on cervical vertebral maturation assessment: a systematic review and meta-analysis. BMC Oral Health. (2025) 25:187. 10.1186/s12903-025-05482-9 39910512 PMC11796225

[32] Han H. Challenges of reproducible AI in biomedical data science. BMC Med Genomics. (2025) 18:8. 10.1186/s12920-024-02072-6 39794788 PMC11724458

[33] Rudin C. Stop Explaining Black Box Machine Learning Models for High Stakes Decisions and Use Interpretable Models Instead. *arXiv [Preprint]*. (2019). 10.48550/arXiv.1811.10154

[34] Price WN Cohen IG. Privacy in the age of medical big data. Nat Med. (2019) 25:37–43. 10.1038/s41591-018-0272-7 30617331 PMC6376961

[35] Varoquaux G Cheplygina V. Machine learning for medical imaging: methodological failures and recommendations for the future. NPJ Digit Med. (2022) 5:48. 10.1038/s41746-022-00592-y 35413988 PMC9005663

[36] Kelly CJ Karthikesalingam A Suleyman M Corrado G King D. Key challenges for delivering clinical impact with artificial intelligence. BMC Med. (2019) 17:195. 10.1186/s12916-019-1426-2 31665002 PMC6821018

[37] Gao Y Zhang P Xie Y Han J Zeng L Ning N. Application of transformers in stomatological imaging: a review. Digital Med. (2024) 10:e24. 10.1097/DM-2024-00001

[38] Motie P Ashkan A Mohammad-Rahimi H Hassanzadeh-Samani S Razzaghi N Behnaz M. Improving cervical maturation degree classification accuracy using a multi-stage deep learning approach. Imaging Sci Dent. (2025) 55:290–301. 10.5624/isd.20250045 41070250 PMC12505443

[39] Kim J Kim T Kim T Kim D-W Ahn B Kim Y-J. Attend-and-refine: interactive keypoint estimation and quantitative cervical vertebrae analysis for bone age assessment. Med Image Anal. (2025) 106:103715. 10.1016/j.media.2025.103715 40769097

[40] Wang P Song W Wang A Xie X Guan X Li T. LM-PCVMNet: pediatric cervical vertebral maturation analysis with deep fusion of landmarks and metadata. Inf Fus. (2027) 138:104699. 10.1016/j.inffus.2026.104699

[41] Kanchanapiboon P Tunksook P Tunksook P Ritthipravat P Boonpratham S Satravaha Y. Classification of cervical vertebral maturation stages with machine learning models: leveraging datasets with high inter- and intra-observer agreement. Prog Orthod. (2024) 25:35. 10.1186/s40510-024-00535-1 39279025 PMC11402886

[42] Lapuschkin S Wäldchen S Binder A Montavon G Samek W Müller K-R. Unmasking clever hans predictors and assessing what machines really learn. Nat Commun. (2019) 10:1096. 10.1038/s41467-019-08987-4 30858366 PMC6411769

[43] Zech JR Badgeley MA Liu M Costa AB Titano JJ Oermann EK. Variable generalization performance of a deep learning model to detect pneumonia in chest radiographs: a cross-sectional study. PLoS Med. (2018) 15:e1002683. 10.1371/journal.pmed.1002683 30399157 PMC6219764

[44] Hooper SM Dunnmon JA Lungren MP Mastrodicasa D Rubin DL Ré C. Impact of upstream medical image processing on downstream performance of a head CT triage neural network. Radiol Artif Intell. (2021) 3:e200229. 10.1148/ryai.2021200229 34350412 PMC8328108

