# Supplementary material for: Does predictive accuracy reflect anatomical attention in CVM staging? evidence from explainable held-out vendor evaluation
Source: Front Med (Lausanne). 2026 Oct 14;13:1971090. doi: 10.3389/fmed.2026.1971090 (PMC13713889; doi:10.3389/fmed.2026.1971090)
Supplement: Supplementary file 1 [file Datasheet1.pdf]

Supplementary File 1

Hyperparameter and Implementation Details

ResNet-50

The ResNet-50 model was initialized with ImageNet-pretrained weights and adapted for six-class CVM classification. All input images were resized to 512 × 512 pixels before training. Grayscale images were used as the network input. The model was optimized using the AdamW optimizer with an initial learning rate of 1×10^{-4}, a weight decay of 1×10^{-4}, and default AdamW momentum coefficients ($\beta_1 = 0.9$, $\beta_2 = 0.999$). No learning-rate scheduler was applied, and a fixed learning rate was maintained throughout training.

Training was performed using a batch size of 32 for 100 epochs. Early stopping was not applied in order to ensure that all preprocessing pipelines were evaluated under an identical experimental framework. Model convergence was assessed using the internal validation set and was considered achieved when the validation accuracy changed by less than 0.01 during the final 10 epochs, while the validation loss reached a stable plateau without progressive divergence from the training loss.

The cross-entropy loss function was used for optimization. No class weighting, oversampling, or undersampling strategies were applied. Data augmentation was applied only to the training set and included random in-plane rotation (±5°) and random translation (up to 2% of the image size in both horizontal and vertical directions). Horizontal flipping, scaling, and shearing were not performed. No augmentation was applied to either the internal validation set or the external validation dataset.

Training was repeated using three independent random seeds (0, 1, and 2) to evaluate result stability. All experiments were conducted using PyTorch 2.5.1 with CUDA 12.1 on an NVIDIA RTX 4080 SUPER (16 GB VRAM). Depending on the preprocessing pipeline, the average training time was approximately 15–25 minutes per seed.

Swin Transformer

The Swin Transformer model was initialized with ImageNet-pretrained weights and trained under the same experimental framework as the ResNet-50 model to ensure fair comparison across preprocessing pipelines. Input images were resized to 512 × 512 pixels and converted to the input format required by the pretrained backbone. The model was optimized using the AdamW optimizer with an initial learning rate of 1×10^{-4}, a weight decay of 1×10^{-4}, and AdamW default coefficients ($\beta_1 = 0.9$, $\beta_2 = 0.999$). No learning-rate scheduler was used during training.

Training was performed with a batch size of 32 for 100 epochs without early stopping. The

same convergence criterion described for ResNet-50 was adopted for all Swin Transformer experiments. The cross-entropy loss function was used for optimization. Class weighting, oversampling, and undersampling were intentionally not applied in order to preserve the natural class distribution and maintain a unified experimental framework across all preprocessing strategies.

Training data augmentation was identical to that used for ResNet-50 and consisted of random in-plane rotation (±5°) and random translation (up to 2% of the image size). No augmentation was applied during internal validation or external testing.

Three independent random seeds (0, 1, and 2) were used for repeated experiments. All Swin Transformer models were trained using PyTorch 2.5.1, timm, and CUDA 12.1 on an NVIDIA RTX 4080 SUPER (16 GB VRAM). The average training time was approximately 25–40 minutes per seed, depending on the preprocessing pipeline.

Supplementary Note

To ensure a fair comparison between preprocessing strategies, all experiments were conducted under a unified training protocol. Unless explicitly specified, identical optimization settings, training schedules, convergence criteria, and evaluation procedures were used across all preprocessing pipelines and both network architectures. The internal validation dataset was used exclusively for monitoring model convergence, whereas the independent external multi-vendor dataset was reserved solely for final performance evaluation.
