# Supplementary material for: Does predictive accuracy reflect anatomical attention in CVM staging? evidence from explainable held-out vendor evaluation
Source: Front Med (Lausanne). 2026 Oct 14;13:1971090. doi: 10.3389/fmed.2026.1971090 (PMC13713889; doi:10.3389/fmed.2026.1971090)
Supplement: Supplementary file 2 [file Datasheet2.pdf]

Supplementary File 2

Class-wise_External_Validation_Performance

Table S2. Class-wise precision, recall, F1-score, and Macro-F1 for the best-performing ResNet-50 (P5) and Swin Transformer (P4) pipelines on the held-out external validation dataset.

Model	Pipeline	CVM stage	n	Precision	Recall	F1-score	Macro-F1
**ResNet-50**	P5	1	20	0.30	0.35	0.32	0.362
		2	20	0.26	0.30	0.28	
		3	21	0.33	0.33	0.33	
		4	70	0.52	0.60	0.56	
		5	52	0.45	0.56	0.50	
		6	17	0.18	0.18	0.18	
**Swin Transformer**	P4	1	20	0.26	0.30	0.28	0.300
		2	20	0.23	0.25	0.24	
		3	21	0.27	0.29	0.28	
		4	70	0.43	0.56	0.49	
		5	52	0.32	0.50	0.39	
		6	17	0.13	0.12	0.12	

Note: Class-wise precision, recall, and F1-score were calculated from the final external-validation results and averaged across the three independent random seeds (0, 1, and 2). Macro-F1 was calculated as the unweighted mean of the six class-wise F1-scores for each model–pipeline combination.

Supplementary File 2

Table S3. Macro-F1 scores for all preprocessing pipelines of ResNet-50 and Swin Transformer on the held-out external validation dataset.

Model	P1	P2	P3	P4	P5	P6
**ResNet-50**	0.323	0.261	0.351	0.327	0.362	0.294
**Swin Transformer**	0.252	0.255	0.231	0.300	0.189	0.243

Note: Macro-F1 was calculated as the unweighted mean of the six class-wise F1-scores, assigning equal weight to each CVM stage. Values were averaged across three independent random seeds (0, 1, and 2). Detailed class-wise precision, recall, and F1-score for the best-performing ResNet-50 (P5) and Swin Transformer (P4) pipelines are provided in Supplementary Table S2.
